# Physiological Response of *Citrus reticulata* Blanco var. Gonggan Seedlings to High-Temperature Stress

**DOI:** 10.3390/life15050806

**Published:** 2025-05-19

**Authors:** Shaoping Wu, Jinyan Liao, Chunxing Ye, Shanyi Chen, Yingshan Wang, Xiaochun Zhang, Junwen Huang, Cong Chen

**Affiliations:** School of Life Sciences, Zhaoqing University, Zhaoqing 526061, China; nhwsp0112@163.com (S.W.); liaojinyan03@163.com (J.L.); y1993106082@163.com (C.Y.); chenshanyi73222@163.com (S.C.); 13976854315@163.com (Y.W.); sweet.zxc@163.com (X.Z.); junwenh@yeah.net (J.H.)

**Keywords:** *Citrus reticulata* Blanco var. Gonggan, high-temperature stress, photosynthesis, peroxidase activity, malondialdehyde

## Abstract

The physiological and biochemical responses of *Citrus reticulata* Blanco var. Gonggan (Gonggan) to high-temperature stress were explored in the present study, offering valuable insights into the growth of this plant in elevated temperature scenarios. Plants were exposed to daytime temperatures of 22 °C, 40 °C, and 45 °C, with corresponding nighttime temperatures of 17 °C, 35 °C, and 40 °C, respectively. Each treatment was administered for 12 h, with a daytime light intensity of 14,400 lux. Key parameters such as the chlorophyll content, peroxidase activity, malondialdehyde content, cytoplasmic membrane permeability, and photosynthetic metrics were assessed. The results showed that the content of malondialdehyde decreased with the increase in temperature, with the highest content at 22 °C. After high-temperature treatment at 40 °C and 45 °C, there was a significant difference (*p* < 0.05) compared with the Gonggan plants treated at 22 °C. Peroxidase activity exhibited an increasing trend as the temperature increased, and there was a significant difference (*p* < 0.05) between the peroxidase activity at 22 °C and 45 °C. Similar trends are displayed for high-temperature stress, stomatal conductance, transpiration rate, and intercellular CO_2_, which initially decreased and then significantly increased. The net photosynthetic rate (Pn) showed a trend of first increasing and then decreasing. When plants were subjected to high-temperature stress at 40 °C, the net photosynthetic rate showed a significant increase compared to the control group at 22 °C, but in a 45 °C stress environment, the Pn showed a decreasing trend. In the experimental group, relative conductivity decreased with the increase in temperature. Meanwhile, Gonggan plants exhibited moderate heat tolerance to short-term or moderate high-temperature stress, primarily through antioxidant and repair mechanisms. However, their heat tolerance was limited under prolonged or extremely high-temperature conditions, characterized by significant membrane damage and photosynthetic inhibition. Overall, Gonggan plants demonstrate moderate heat tolerance, making them suitable for intermittent high-temperature environments rather than prolonged extreme heat conditions. These findings provide a foundation for understanding the adaptive strategies of Gonggan plants and their cultivation in high-temperature settings.

## 1. Introduction

*Citrus reticulata* Blanco var. Gonggan (Gonggan) is a well-known citrus fruit from Deqing District in Zhaoqing City. It is also known as Emperor’s Orange due to its designation as a tribute during the Northern Song Dynasty [1]. Gonggan is rich in phytochemicals that are beneficial to human health, such as flavonoids, vitamin C, carotenoids, limonoids, and terpenes. Flavonoids in Gonggan have antibacterial properties [2,3]. During their growth and development, plants may encounter extreme conditions, such as high salinity, frost, drought, and diseases, collectively termed adversity, which significantly limit agricultural production as they can hinder crop distribution, diminish yield and quality, and potentially lead to crop failure [4,5]. Recently, the increased frequency and intensity of high-temperature stress due to global warming have emerged as critical factors affecting plant growth and development [6]. Therefore, studying the effects of high-temperature stress on the growth, development, and physiological characteristics of Gonggan is essential for enhancing its stress resistance and developing resistant varieties.

High-temperature stress is a major form of environmental stress that limits plant growth and metabolism. This phenomenon typically involves temperatures reaching a critical threshold and remaining elevated for an extended duration, causing irreversible damage to plant growth and development. Both short-term and prolonged exposure to high temperatures can induce physiological and biochemical changes, disrupting processes such as photosynthesis, water metabolism, endogenous hormone levels, and metabolite concentrations. These disruptions can significantly reduce crop yields [7]. Research has established a strong link between rice yields and average minimum temperatures during the dry season (January to April), with a 1 °C increase in minimum temperature resulting in a 10% decrease in grain yield, while the impact of maximum temperatures is less pronounced. Rising nighttime temperatures due to global warming have also been associated with lower rice yields [8]. Additionally, drought and extreme heat have led to a 9–10% reduction in national grain production [9]. Studies have shown that a 1 °C rise in temperature correlates with a 10% decrease in rice yield and a 3–4% decline in wheat yield [10,11]. Moreover, daily temperatures above 30 °C can result in a 1% yield reduction in corn and barley [12]. Heat stress further affects respiration and photosynthesis, shortening life cycles and diminishing plant productivity [13]. Generally, high-temperature stress adversely impacts metabolite biosynthesis [14]. Heat stress in tomato plants leads to decreased peroxidase (POD) activity, which may help these plants to adapt to higher temperatures [15]. High-temperature stress in Nanfeng Tangerine plants led to an initial increase followed by a decrease in the activities of the SOD, POD, and CAT enzymes in Nanfeng Tangerine plant leaves [16]. During heat stress, levels of essential hormones such as abscisic acid, ethylene, and salicylic acid increase, while gibberellins, cytokinins, and auxins decrease, contributing to premature aging [17,18,19]. Thus, heat stress significantly impacts plant growth, development, and physiological functions.

Plant leaves can, to some extent, reflect the physiological adaptability of plants and serve as a basis for studying stress resistance. Various physiological indicators were evaluated in the present study, including the chlorophyll content, POD activity, malondialdehyde (MDA) content, cytoplasmic membrane permeability, and photosynthetic metrics, in Gonggan plants under high-temperature stress conditions. While numerous studies have documented the adverse effects of high-temperature stress on plant growth [16,20,21,22,23], research focusing specifically on Gonggan (Citrus reticulata cv. Gonggan) remains notably scarce.

This study examines the impact of high-temperature stress on key physiological parameters in Gonggan leaves, including the chlorophyll content, peroxidase (POD) activity, malondialdehyde (MDA) content, cytoplasmic membrane permeability, and photosynthetic efficiency. Furthermore, it elucidates the response mechanisms of the photosynthetic and antioxidant systems under varying degrees of thermal stress. The findings provide critical theoretical insights and practical guidance for enhancing Gonggan’s heat resistance during summer, thereby mitigating growth-related thermal damage.

## 2. Materials and Methods

### 2.1. Test Materials

**Figure 1:**
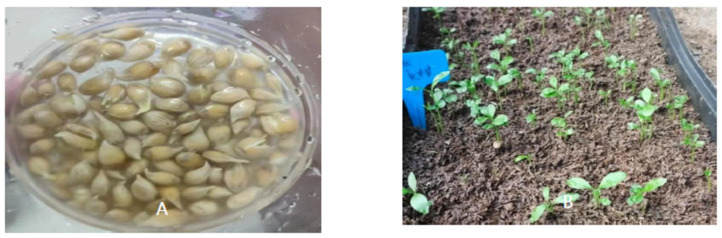
Young Gonggan seedlings. (**A**) Gonggan seed; (**B**) Gonggan seedling.

A total of 517 Gonggan seeds were cultivated, yielding 267 germinations, equating to a germination rate of 51.6%. Of these, 81 buds grew into seedlings, representing a seedling rate of 30.3%. All tribute orange plants were planted in the Biological Park at Zhaoqing University (Figure 1).

### 2.2. Determination of Physiological Parameters in Gonggan Seedlings

**Figure 2:**
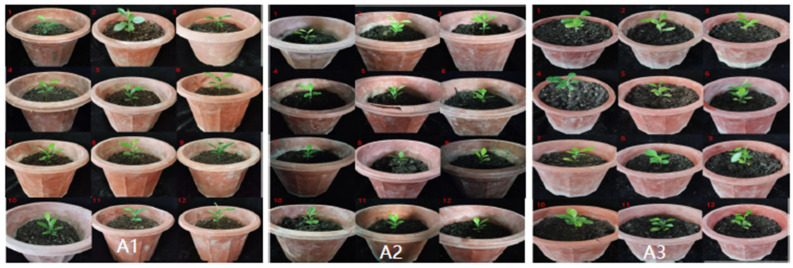
Three replicate samples of transplanted Gonggan seedlings (**A1**), (**A2**), and (**A3**).

Thirty-six uniformly growing Gonggan seedlings were selected and divided into three groups of 12 plants each (Figure 2). The width, length, and area of the top three leaves were measured. Additionally, the plant height, number of main stem leaves, and the dimensions (length, width, and area) of Gonggan leaves were recorded (Table 1). Plant height was measured from the base to the highest point of the main stem using a meter ruler. The total leaf count was recorded manually, and the leaf area was measured with a leaf area meter.

#### 2.2.1. Experimental Design

Gonggan Demonstration Park, located in Gemu Village, Xinwei Town, Deqing County, Guangdong Province, was identified as having an optimal growth temperature of 23.0–31.0 °C for Gonggan, with germination starting at 12.5 °C and ceasing when temperatures reach 35.0–37.0 °C. To ensure safe winter cultivation, temperatures should not fall below 5.0 °C.

Plants were cultivated in a Ningbo Jiangnan GXZ-500C light incubator (Shanghai Yinze Instrument Equipment Co., Ltd., Shanghai, China), with daytime temperatures set at 22, 40, and 45 °C. Twelve tribute orange plants were chosen from each temperature group. Daytime light intensity was maintained at 14,400 lux for 12 h, while nighttime temperatures were adjusted to 5 °C lower than the daytime temperatures (17, 35, and 40 °C) for the same duration, with relative humidity at 75%. Various indicators, including the chlorophyll a (Ca) and b (Cb) contents, POD activity, MDA content, cytoplasmic membrane permeability, and photosynthetic metrics, among others, were assessed. The physiological response of most plants to high-temperature stress involves an initial increase in POD activity, which then decreases with prolonged stress, peaking at 24 h [24]. To ensure enzyme activity, a 24 h high-temperature stress period was applied in this study.

#### 2.2.2. Detection of Membrane Permeability in Gonggan Leaves

The permeability of the cytoplasmic membrane was evaluated using the conductivity method [25]. After a 24 h high-temperature stress treatment on Gonggan seedlings, several similarly aged leaves were selected, washed, and dried in distilled water. Subsequently, 36 leaf discs were extracted from these leaves using an 8 cm puncher and divided into two groups. The leaf discs in the experimental group were placed in a clean beaker with 10 mL of deionized water, shaken vigorously at room temperature, and allowed to stand for 30 min before the conductivity of the soaking solution (R1) was measured. The samples were then boiled in a water bath at 100 °C for 10 min, and the conductivity of the boiling solution (R2) was measured after cooling to room temperature, with this process repeated thrice. The control group was wrapped in moist gauze and kept at room temperature. After treatment, the leaves were washed and dried with filter paper, and 10 mL of deionized water was added to the beaker, ensuring the blades were submerged, with three repetitions for each treatment. Cell membrane damage was quantified using relative conductivity, calculated as relative conductivity (%) = [conductivity value of soaking solution/conductivity value of boiling solution] × 100%.

#### 2.2.3. Determination of Chlorophyll Content

The chlorophyll content was measured using a lengthy anhydrous ethanol extraction method [21]. Optical density was measured at 665 nm and 649 nm, with a 95% ethanol solution as a blank. The concentrations of chlorophyll a, chlorophyll b, and total chlorophyll were calculated using the following formulas: Ca = 13.95 OD_665_ − 6.88 OD_649_; Cb = 24.96 OD_649_ − 7.32 OD_665_; and total chlorophyll concentration = C_a_ + C_b_.

#### 2.2.4. Determination of MDA Content

The MDA content was measured using the thiobarbituric acid method [26]. The MDA content was calculated using the following formula:

MDA content (μ moL/g) = [C × V]/m × 1000

where C = 6.45 (OD_532_ − OD_600_) − 0.56OD_450_; C represents MDA concentration (μ mol/L); V is the volume of the extraction solution (mL); and m is the fresh weight of plant tissue (g).

#### 2.2.5. Determination of POD Activity

POD activity was evaluated using the guaiacol colorimetric method [27]. The enzyme activity was calculated using the following formula:

POD activity = Δ A_470_ × VT/(m × Vs × 0.01×Δ t)

where ∆ A_470_ represents the change in absorbance during the initial reaction rate stage; m is the mass of plant material (g); Δ t is the reaction time (min) corresponding to ∆ A_470_; VT is the total volume of the extracted enzyme solution (mL); Vs is the volume of enzyme solution used for measurement (mL); and 0.01 is a unit of enzyme activity based on Δ A_470_.

#### 2.2.6. Determination of Photosynthetic Metrics

The net photosynthetic rate (Pn), stomatal conductivity (Gs), intercellular carbon dioxide (CO_2_) concentration (Ci), and transpiration rate (Tr) were measured in leaves at the 2nd, 3rd, and 4th levels of the main stem, with consistent light exposure. Measurements were taken using the HED-GH30 plant photosynthesis instrument. Each temperature treatment involved 12 plants, totaling 36 plants across three temperature conditions. Leaves were exposed to 14,400 lux for 30 min in a light incubator to activate the photosynthetic system, using an open-air path for measurements.

### 2.3. Statistical Analysis

The World Programming System (WPS) was utilized to organize data for various indicators. Data were compared using one-way ANOVA and an independent sample *t*-test with SPSS 27 software. The line and bar charts were both generated using SPSS.27.

## 3. Results

### 3.1. Effect of High-Temperature Stress on MDA Content

**Figure 3:**
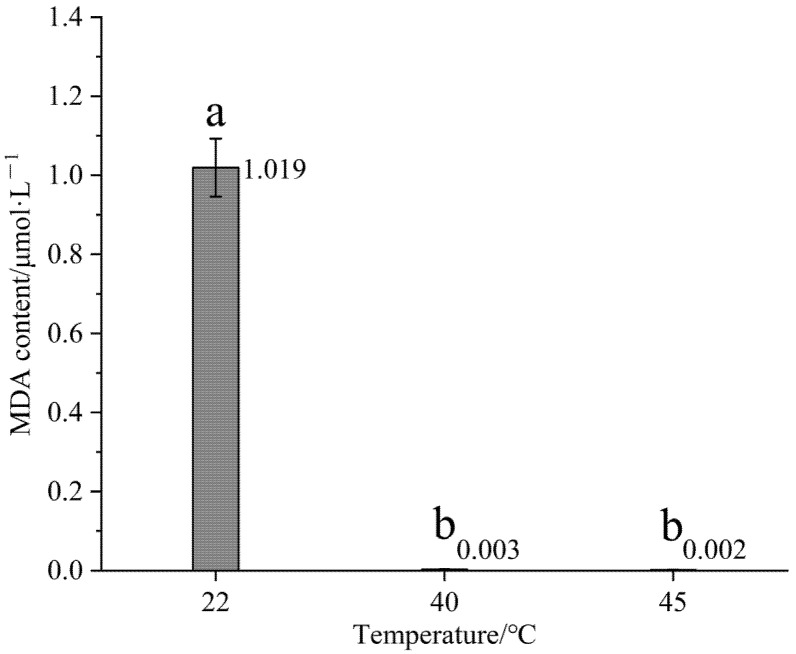
Effect of 40 and 45 °C temperature treatments on MDA content of Gonggan seedlings. Different lowercase letters indicate significant differences (*p* < 0.05).

Under varying temperature conditions, the MDA content in Gonggan seedlings exhibited significant differences across the population (*p* < 0.01). Notably, there were also significant differences among all temperatures (*p* < 0.05). As shown in Figure 3, the MDA content decreased with the increase in temperature, peaking at 22 °C. After exposure to elevated temperatures of 40 and 45 °C, a significant difference was observed between Gonggan plants subjected to 40 °C and those at 22 °C (*p* < 0.05).

### 3.2. Effect of High-Temperature Stress on Chlorophyll Content

**Figure 4:**
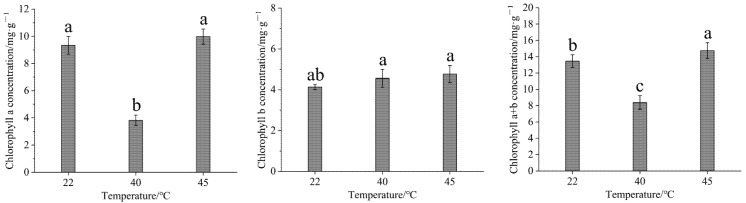
Effect of high-temperature stress on chlorophyll content in Gonggan seedlings. Different lowercase letters indicate significant differences (*p* < 0.05).

After high-temperature stress, the chlorophyll a concentration in Gonggan leaves initially decreased and then increased. Conversely, chlorophyll b did not significantly increase, while the chlorophyll a + b concentration exhibited a similar “decrease then increase” trend (Figure 4). A significant difference in the chlorophyll a concentration was observed between 40 °C and both 22 and 45 °C (*p* < 0.05). There was no significant difference in the chlorophyll a concentration between 22 and 45 °C (*p* > 0.05). However, a significant difference in the chlorophyll b concentration was found between 22 and 45 °C (*p* < 0.05) and in the chlorophyll a + b concentration across the three temperature conditions (*p* < 0.05).

### 3.3. Effects of High-Temperature Stress on Photosynthetic Metrics

**Figure 5:**
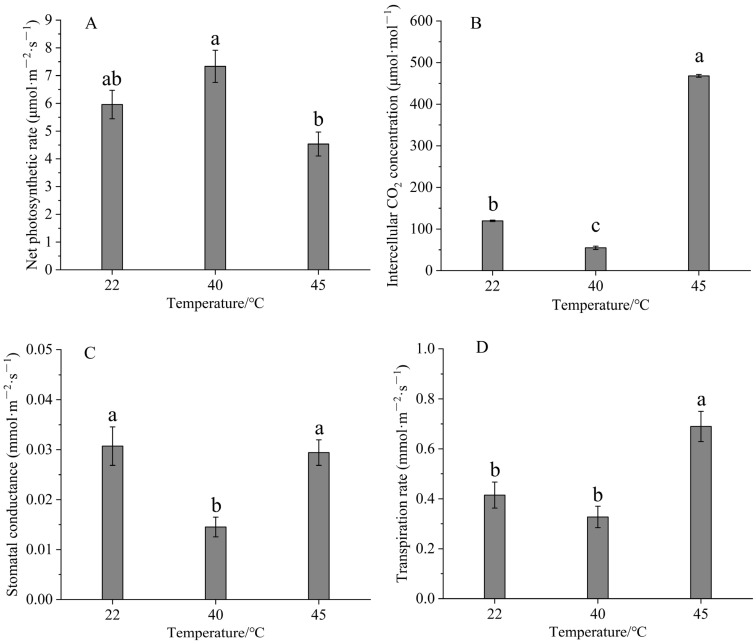
Effects of high-temperature stress on photosynthetic metrics of Gonggan leaves. (**A**) Net photosynthetic rate. (**B**) Intercellular CO_2_ concentration. (**C**) Stomatal conductance. (**D**) Transpiration rate. Different lowercase letters indicate significant differences (*p* < 0.05).

Photosynthetic parameters included the net Pn (μ molm^−2^ s^−1^), Gs (mmolm^−2^ s^−1^), Ci (μmolmol^−1^), and Tr (mmolm^−2^ s^−1^). The measurement results are shown in Figure 5.

Compared with the control group (22 °C), the Pn was significantly increased under high-temperature stress (40 °C) but demonstrated a downward trend under extreme stress (45 °C). The Gs, Tr, and Ci also initially decreased before significantly rising during high-temperature stress, with peak values at 45 °C, while the Tr reached its lowest value at 40 °C.

### 3.4. Effect of High-Temperature Stress on the Cytoplasmic Membrane Permeability

**Figure 6:**
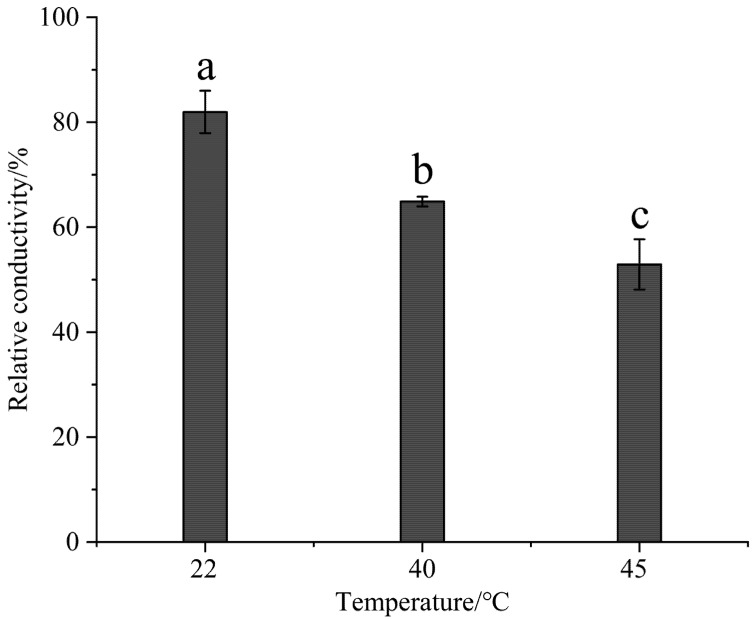
Effects of high-temperature stress on the cytoplasmic membrane permeability of Gonggan leaves. Different lowercase letters indicate significant differences (*p* < 0.05).

Significant differences were noted among the experimental groups at different temperatures (*p* < 0.01) and between the control groups (*p* < 0.01). The relative conductivity of the experimental group reduced dramatically with the rise in temperature, while that of the control group slightly increased (Figure 6).

### 3.5. Effect of High-Temperature Stress on POD Activity

**Figure 7:**
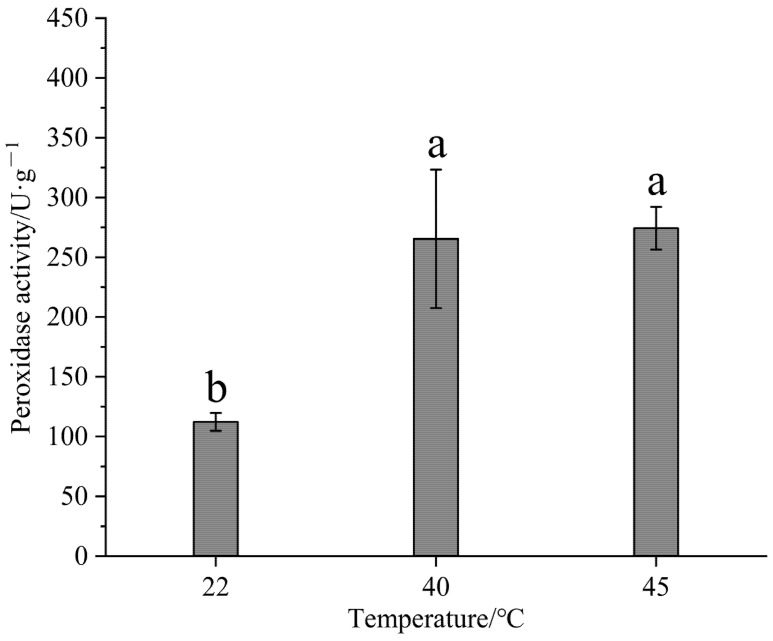
Effects of high-temperature stress on peroxidase activity in Gonggan leaves. Different lowercase letters indicate significant differences (*p* < 0.05).

POD is a crucial enzyme in the plant antioxidant system, primarily catalyzing the conversion of hydrogen peroxide (H_2_O_2_) into water and oxygen. This reaction reduces the accumulation of ROS, protecting cell membranes, proteins, and nucleic acids from oxidative damage. High temperatures can significantly increase ROS, including superoxide anions and H_2_O_2_, within plant cells. The increase in POD activity acts as a defense mechanism, allowing plants to actively eliminate ROS and maintain redox balance. Our results showed that POD activity increased with temperature, with a significant difference noted between 22 and 45 °C (*p* < 0.05) (Figure 7).

## 4. Discussion

Elevated temperatures adversely affect citrus plants in two main ways: physiological metabolic disruption and organ damage. When temperatures exceed 35 °C, the stomatal closure mechanism of citrus leaves becomes unbalanced, causing transpiration rates to exceed the water absorption capacity of the roots, leading to water stress symptoms such as leaf wilting and curling leaves. Furthermore, when fruit surface temperatures surpass 38 °C, irreversible damage to the skin cells occurs, resulting in sunspots that significantly reduce market value. Research shows that the effects of high temperatures and drought on citrus primarily manifest through leaf wilting, the formation of small, scorched fruits, and stunted growth [28]. Therefore, exploring the mechanisms of high-temperature stress and the roles of heat response signaling pathways and stress resistance genes can deepen our understanding of plant stress biology. These insights can guide the breeding of heat-resistant varieties, improve cultivation practices (such as shading and cooling techniques and the use of anti-transpiration agents), and enhance crop resilience to extreme weather. This research is of strategic importance in the realm of climate change as it aims to secure food security, promote sustainable agricultural development, and offer scientific guidance for ecological restoration and the development of stress-resistant germplasm resources.

MDA levels in stressed plants are a reliable indicator of lipid peroxidation in plant cells [29]. MDA serves as an indicator of plant cell membrane damage. Stressors such as high temperatures, drought, and salinity lead to the accumulation of reactive oxygen species (ROS) in the cells, resulting in membrane lipid peroxidation and increased MDA production. Typically, MDA levels rise under stress, indicating cell membrane damage [30]. Research on the heat tolerance of seedlings has revealed that the MDA content exhibits varying patterns depending on the plant variety, with a noted decrease in MDA levels. This reduction may be attributed to significant damage to leaf cells caused by high temperatures, resulting in the partial breakdown of MDA, or it could be linked to the plant’s internal protective mechanisms that facilitate MDA degradation. Additionally, it is plausible that the plants activate their antioxidant systems, enhancing the activity of enzymes such as superoxide dismutase (SOD), peroxidase (POD), and catalase (CAT) to eliminate excess reactive oxygen species (ROS), thereby mitigating membrane lipid peroxidation and contributing to the decline in MDA levels. Furthermore, the response of the MDA content to stress may differ based on the materials involved under high-temperature conditions. Variations in stress duration or intensity could lead to an initial increase followed by a decrease in MDA levels, or the plants might quickly recover after a brief stress period, resulting in lower MDA content [31]. These findings are similar to those pertaining to the heat resistance of bitter gourd and Helleborus orientlis [32,33]. The POD activity increases with temperature (5–30 °C), with the optimal temperature for activity being around 25–30 °C. Beyond 30 °C, POD activity decreases with the increase in temperature, ultimately reaching an inactive state at 70 °C [34]. These results are consistent with the research of Chang Cuifang et al. [35] on Hebes plants. Ye Boyu et al. [36] studied the effects of high-temperature stress on the photosynthesis and antioxidant system of *Carya cathayensis* and found that the contents of superoxide dismutase (SOD), catalase (CAT), and peroxidase (POD) increased with the increase in temperature when treated at 25 °C to 35 °C but significantly decreased at 40 °C.

It was found in the present study that after exposure to high-temperature stress, the concentration of chlorophyll a in Gonggan leaves initially decreased before increasing again. In contrast, chlorophyll b did not show a significant increase. Notably, chlorophyll a + b followed a similar trend of first decreasing and then increasing. This result is consistent with those of Yang Li et al. in studying the heat tolerance of cucumber [37]. This is also consistent with Xue Jianping et al.’s study on the photosynthetic parameters of Pinellia ternata [38]. This phenomenon may be attributed to the substantial effect of temperature on enzyme activity, as chlorophyll synthesis relies on various enzymatic reactions. High-temperature stress typically leads to a decline in the chlorophyll content due to its impact on the intermediate products of chlorophyll biosynthesis. Furthermore, elevated temperatures increase ROS production, making leaves more susceptible to oxidative damage [39]. Chlorophyll b primarily functions as an auxiliary pigment, and its molecular structure is relatively stable, resulting in slower degradation under light-induced damage conditions, which explains the minimal changes in its concentration.

Key indicators such as the Pn, Gs, Ci, and Tr reflect a plant’s adaptability to the environmental conditions during the photosynthetic diurnal cycle. High temperature significantly affects plant growth. In most plants, the reduction in the Pn under high-temperature stress is mainly due to increased transpiration rates rather than water supply issues, resulting in a water deficit that causes stomatal or non-stomatal limitations [40]. Unlike in this study, Wu Xiaming et al. found that the net photosynthetic rate, stomatal conductance, and transpiration rate of strawberry seedlings showed a decreasing trend under high-temperature stress [41]. In the study of photosynthesis in *Carya cathayensis* by Ye Boyu et al. [36], it was shown that under mild stress, the Pn showed a significant upward trend compared to the control group (normal temperature), consistent with our findings. Under high-temperature stress, Pn began to decrease.

The Ci declines before subsequently rising. During the early stages of high-temperature stress, the Gs drops, limiting CO_2_ entry into the leaves. Meanwhile, the Pn may remain stable, causing the plant to continuously consume intercellular CO_2_, which significantly lowers the Ci. However, as high-temperature stress extends into the later stage, the photosynthetic system is severely damaged, leading to a notable decline in the Pn and CO_2_ consumption. Even if stomatal conductance improves, the reduced photosynthetic capacity results in lower intercellular CO_2_ uptake compared to the supply, leading to an increase in the Ci.

High temperatures can increase water loss from plant leaves. To counter this loss, plants reduce transpiration by closing their stomata, which decreases stomatal conductance and serves as a protective mechanism for plants experiencing high-temperature stress. Plants may modify their physiological processes as they gradually acclimatize to elevated temperature conditions. For instance, plants may enhance their water retention capabilities by producing osmoregulatory compounds, which help alleviate the impacts of water stress and enable appropriate stomatal opening. Temperature significantly affects transpiration rates. The kinetic energy of water molecules increases with the rise in temperature, increasing the water vapor pressure gradient on the leaf surface. This results in an accelerated diffusion of water from the leaf to the atmosphere, continuously increasing the transpiration rate.

Relative conductivity is a crucial metric for assessing plant cell membrane integrity, with elevated values signifying greater membrane damage and heightened electrolyte leakage. Stress factors such as high temperature, drought, and salinity can lead to membrane lipid peroxidation or membrane protein denaturation, compromising membrane structure and raising electrical conductivity. This metric is often used to evaluate plant stress resilience. The decrease in relative conductivity at elevated temperatures may result from the increased activity of SOD, CAT, and POD, which mitigates the effects of ROS and reduces oxidative damage to the membranes. Both relative conductivity and MDA levels indicate membrane lipid peroxidation, thereby indirectly reflecting the extent of thermal injury. The simultaneous reduction in relative conductivity and MDA suggests less membrane lipid peroxidation. The experimental group showed significantly better membrane stability than the control group at high temperatures, indicating strong heat tolerance. The cell membrane effectively resists high-temperature stress and minimizes damage through adaptive responses or protective mechanisms. This finding should be corroborated by additional indicators, such as chlorophyll fluorescence (such as Fv/Fm), to assess potential damage to the photosynthetic apparatus and by evaluating the expression levels of heat shock proteins such as HSP70 and HSP90 to confirm the heat stress response.

## 5. Conclusions

In this study, the chlorophyll content, POD activity, MDA content, cytoplasmic membrane permeability, and photosynthetic parameters in the leaves of Gonggan seedlings were measured under high-temperature stress at 22, 40, and 45 °C. In response to high-temperature stress, the content of malondialdehyde decreased, and there was no significant difference in the malondialdehyde content after 40 °C. The concentration of chlorophyll a + b first decreased and then increased, and there were significant differences in the chlorophyll a + b concentration between the three temperatures. After high temperature treatment, the relative conductivity of the leaves gradually decreased, there was a significant difference in conductivity after three temperature treatments, and the POD activity increased with the increase in temperature. The POD activity at 22 degrees Celsius was significantly different from that at 40 and 45 °C, while there was no significant difference in POD activity between the 40 and 45 °C treatments. Under high-temperature stress, the stomatal conductance (Gs), transpiration rate (Tr), and intercellular CO_2_ concentration (Ci) of leaves first decreased and then increased, while the net photosynthetic rate (Pn) increased and then decreased. This study revealed the physiological regulation mechanism of Gonggan plants at high temperatures, confirming that Gonggan plants can resist high-temperature damage through physiological regulation.

## Figures and Tables

**Table 1:** Determination of plant height and leaf area in Gonggan seedlings.

Characters	No	Min	Max	Ave	STDEV
Width	36	9.00	17.00	13.08	2.38
Length	36	39.00	105.00	68.75	14.20
Number of leaves	36	6.00	14.00	8.67	1.57
Plant height	36	5.00	8.80	6.14	0.95
Leaf area	36	1.13	4.55	2.59	0.90

## Data Availability

Data are contained within the article. The original contributions presented in the study are included in the article; further inquiries can be directed to the corresponding author.
